# Transcriptome Analysis of Particulate Matter 2.5-Induced Abnormal Effects on Human Sebocytes

**DOI:** 10.3390/ijms231911534

**Published:** 2022-09-29

**Authors:** Hye-Won Na, Hyun Soo Kim, Hyunjung Choi, Nari Cha, Young Rok Seo, Yong Deog Hong, Hyoung-June Kim

**Affiliations:** 1 Research and Innovation Center, AMOREPACIFIC, Yongin 17074, Korea; 2 Department of Life Science, Institute of Environmental Medicine, Dongguk University Biomedi Campus, Goyang 10326, Korea

**Keywords:** particulate matter 2.5, transcriptome analysis, human sebocytes, ingenuity pathway analysis, lipid peroxidation

## Abstract

Particulate matter 2.5 (PM_2.5_), an atmospheric pollutant with an aerodynamic diameter of <2.5 μm, can cause serious human health problems, including skin damage. Since sebocytes are involved in the regulation of skin homeostasis, it is necessary to study the effects of PM_2.5_ on sebocytes. We examined the role of PM_2.5_ via the identification of differentially expressed genes, functional enrichment and canonical pathway analysis, upstream regulator analysis, and disease and biological function analysis through mRNA sequencing. Xenobiotic and lipid metabolism, inflammation, oxidative stress, and cell barrier damage-related pathways were enriched; additionally, PM_2.5_ altered steroid hormone biosynthesis and retinol metabolism-related pathways. Consequently, PM_2.5_ increased lipid synthesis, lipid peroxidation, inflammatory cytokine expression, and oxidative stress and altered the lipid composition and expression of factors that affect cell barriers. Furthermore, PM_2.5_ altered the activity of sterol regulatory element binding proteins, mitogen-activated protein kinases, transforming growth factor beta-SMAD, and forkhead box O3-mediated pathways. We also suggest that the alterations in retinol and estrogen metabolism by PM_2.5_ are related to the damage. These results were validated using the HairSkin^®^ model. Thus, our results provide evidence of the harmful effects of PM_2.5_ on sebocytes as well as new targets for alleviating the skin damage it causes.

## 1. Introduction

Atmospheric pollutants from global industrialization cause serious human health problems. In 2005, the World Health Organization (WHO) published air quality guidelines regarding the major health-damaging air pollutants, including particulate matter (PM), ozone, nitrogen dioxide, and sulfur dioxide. In 2019, more than 90% of the world’s population lived in areas with concentrations of particulate matter 2.5 (PM_2.5_) exceeding the air quality guideline (10 μg/mL) [1].

Particulate matter (PM) is classified according to its aerodynamic diameter; PM_2.5_ has an aerodynamic diameter of <2.5 μm. PM_2.5_ contains various chemicals, including metal elements (arsenic, cadmium, lead, etc.), ionic components (ammonium, nitrate, sulfite ions, etc.), and carbonaceous fractions (organic carbon, elemental carbon, polycyclic aromatic hydrocarbons [PAHs], etc.) [2,3]. The small size of PM_2.5_ allows them to enter bronchioles, alveoli, and blood, through which they can further affect other organs [4,5]. PM can penetrate the barrier-disrupted epidermis by tape-stripping and can enter hair follicles regardless of barrier damage [6,7].

The human sebaceous gland, a skin appendage, together with the associated hair follicle and arrector pili muscle, collectively form the pilosebaceous unit [8,9]. The main function of the sebaceous gland in humans is the production and holocrine secretion of sebum, which regulates lipid homeostasis, lubricates the skin, and acts as a defense against various environmental agents [10]. Sebaceous glands also regulate skin homeostasis by controlling cutaneous steroidogenesis, androgen synthesis, and neuroendocrine function [8,11]. Abnormal differentiation of sebaceous glands, increases in lipid synthesis, and variation in lipid composition can cause barrier damage and skin problems, such as seborrheic dermatitis and acne [8,12,13]. Furthermore, the dysregulation of the sebaceous gland might be involved in the development of other inflammatory skin diseases, such as psoriasis and atopic dermatitis [14,15]. Exposure to PM causes skin damage and is associated with several skin diseases by inducing skin barrier damage and alterations in cell proliferation, differentiation, and lipid composition [16]. Moreover, PM increases inflammation and induces oxidative stress via the generation of reactive oxygen species (ROS), which can cause damage to lipids, proteins, and DNA and can induce apoptosis and senescence. Although there are other cell types in the skin, including various immune cells, melanocytes, and sebocytes, most of these effects are focused on epidermal keratinocytes and dermal fibroblasts. Therefore, it is necessary to understand the effects of PM on other skin cell types, especially for sebaceous glands attached to hair follicles, because they are one of the skin penetration routes of PM_2.5_. Herein, we evaluated the effect of PM_2.5_ on SZ95 sebocytes which are the immortalized human sebaceous gland cell line with simian virus-40 large T antigen and retain fundamental characteristics of normal human sebocyte including differentiation and lipid synthesis [17,18,19] and validated the abnormal effects of PM_2.5_ on the HairSkin^®^ model.

## 2. Results

### 2.1. Differentially Expressed Genes (DEGs) and Gene Ontology (GO)-Based Gene-Set Enrichment Analysis of SZ95 Sebocytes after PM_2.5_ Treatment

Based on the results of the cell viability test (Figure S1), we determined the optimal treatment concentration of PM_2.5_. From RNA-sequencing (RNA-seq) data, we identified that the expression of 381 genes (−2 > fold changes > 2; *p*-value < 0.05) was significantly changed in PM_2.5_-treated experimental groups compared with the control SZ95 sebocytes. Treatment with PM_2.5_ resulted in the upregulation of 188 genes and the downregulation of 193 genes.

The top 10 enriched GO terms for biological processes and molecular functions are shown in Figure S2. The most highly enriched molecular functions were related to binding, including protein binding, ion binding, cation binding, and metal ion binding. In addition, oxidoreductase activity was significantly enriched. Oxidoreductases such as aldo-keto reductase (AKR) and cytochrome P450 (CYP450) are related to the metabolism of chemicals, including steroids [20]. Thus, the GO enrichment analysis indicated that PM_2.5_ -induced functional changes are caused by various components of heterogenic PM_2.5_.

### 2.2. Kyoto Encyclopedia of Genes and Genomes (KEGG) Terms and Canonical Pathway Prediction Analysis

**Figure 1:**
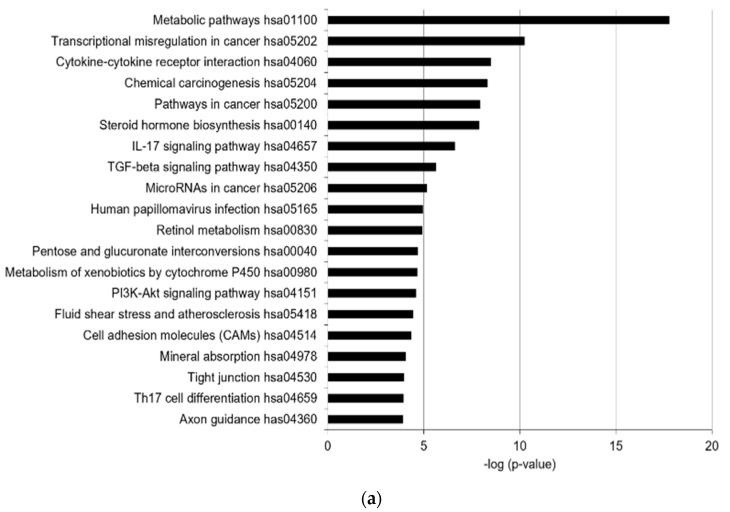

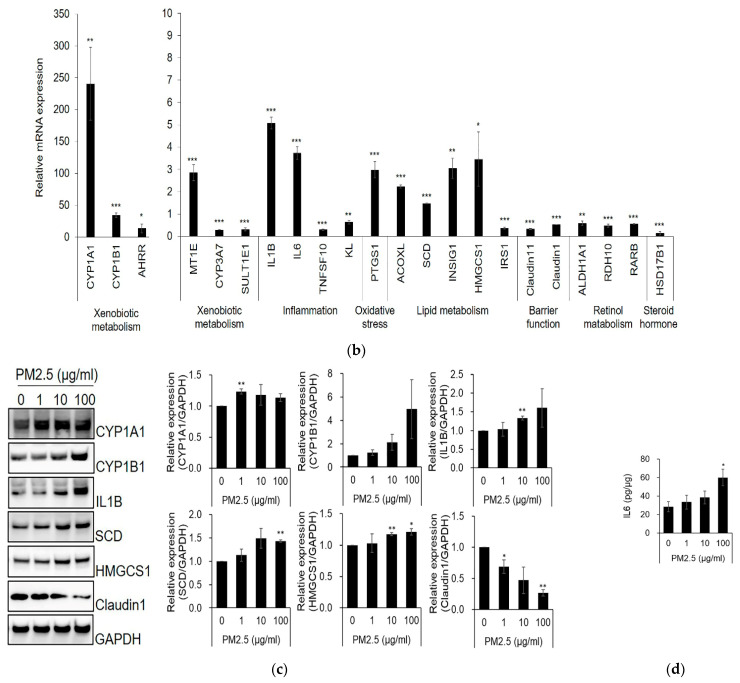
PM_2.5_ alters the expression of xenobiotic metabolism, inflammatory response, oxidative stress, lipid metabolism, and cell barrier-related factors. Transcriptome analysis with RNA-seq was performed using SZ95 sebocytes treated with PM_2.5_ (100 μg/mL) and a control group. The top 20 most prevalent KEGG terms (**a**) were obtained from pathway enrichment analysis of differentially expressed genes (DEGs; |fold changes| > 2, *p*-value < 0.05) in SZ95 sebocytes with PM_2.5_ treatments. Bars represent −log10 (*p*-value). *p*-values were calculated by modified Fisher’s exact tests. (**b**) RT-qPCR was performed to analyze the mRNA expression of DEGs. (**c**) The expression levels were analyzed by SDS-PAGE and immunoblotting for the indicated antibodies. The relative expression levels of proteins were quantified using the Image J program. (**d**) IL6 secretion was analyzed by ELISA and normalized to the total protein. Data were analyzed by Student’s *t*-test (* *p* < 0.05, ** *p* < 0.01, *** *p* < 0.001 vs. untreated control; n = 3).

In total, 55 KEGG pathways were enriched (false discovery rate [FDR] < 0.05), with the top 20 KEGG terms shown in Figure 1a. In addition, 18 canonical pathways were identified (−2 > z-score > 2) by Ingenuity Pathway Analysis (IPA), with the top 10 listed in Table 1. The highly predicted KEGG pathways were metabolic pathways related to steroid hormone biosynthesis, retinol metabolism, pentose and glucuronate interconversions, and the metabolism of xenobiotics by CYP450 (Figure 1a). Xenobiotics produced by CYP450 are known to be involved in the toxicity of PM_2.5_ [16,21]. Steroid hormone biosynthesis and retinol metabolism regulate the functions of sebaceous glands, such as lipid synthesis [8,22]. Inflammatory response-related pathways, such as cytokine–cytokine receptor interaction and the IL-17 signaling pathway, were enriched. In addition, cell adhesion-related pathways, including tight junctions and cell adhesion molecules, were significantly enriched, implying that PM_2.5_ affects barrier function. The transforming growth factor beta (TGFβ) and PI3K-Akt signaling pathways were predicted to be enriched by PM_2.5_. Via canonical pathway analysis, cholesterol biosynthesis-related pathways, including the superpathway of cholesterol biosynthesis, three cholesterol biosynthesis (I, II, III), and superpathway of geranylgeranyl diphosphate biosynthesis I, were significantly enriched (Table 1). The xenobiotic metabolism aryl hydrocarbon receptor (AHR) signaling pathway and estrogen-dependent breast cancer signaling pathway related to steroid hormone metabolism were enriched, consistent with the KEGG analysis results. Moreover, endothelial nitric oxide synthase (eNOS) signaling, which is associated with oxidative stress, was also enriched. Thus, we identified that PM_2.5_ mainly alters the levels of genes associated with xenobiotic metabolism, inflammatory responses, oxidative stress, lipid metabolism, steroid hormone biosynthesis, retinol metabolism, and cell adhesion. Among the DEGs (−2 > fold changes > 2, and *p*-value < 0.05), the genes related to the enriched pathways are summarized in Table S1. The expression of significantly altered genes was confirmed by real-time quantitative reverse-transcription polymerase chain reaction (RT-qPCR), Western blotting analysis, and enzyme-linked immunosorbent assay (ELISA) (Figure 1b–d and Figure S3).

### 2.3. Upstream Regulator Analysis and Disease and Biological Function Prediction by IPA

We analyzed upstream regulators by IPA; the top 10 predicted activated and inhibited upstream regulators in SZ95 sebocytes by PM_2.5_ are listed in Table 2. As a result, sterol regulatory element binding transcription factor (SREBF, which encodes sterol regulatory element binding protein, SREBP) signaling pathway-associated factors were predicted as upstream regulators. SREBF1, SREBF2, and SREBP cleavage-activating protein (SCAP) were activated, and INSIG1, which inhibits SREBP-mediated signaling pathways, was inhibited. Additionally, mitogen-activated protein kinase (MAPK)-mediated signaling pathway-related factors MAPK7 and MAP2K5 were predicted to be activated by upstream regulators. SREBPs regulate lipid homeostasis and inflammation [23,24]. Moreover, MAPK7 and its upstream activator, MAP2K5, are also known to control lipid metabolism in small cell lung cancer cells [25]. In contrast, TGFβ signaling pathway-related factors, such as TGFβ1 and SMAD3, and forkhead box O3 (FoxO3) were all predicted to be inhibited by upstream regulators. TGFβ-SMAD and FoxO3 signaling were associated with the differentiation and lipid synthesis of sebocytes [26,27]. These results imply that PM_2.5_ affects sebocyte differentiation, lipid synthesis, and inflammation by altering various signaling pathways. Furthermore, IPA predicted diseases and biological functions in PM_2.5_-treated SZ95 sebocytes (Table 3). Lipid metabolism and organismal survival were predicted to be PM_2.5_-associated biological functions, and dermatological diseases and cancer were predicted to be associated diseases.

**Figure 2:**
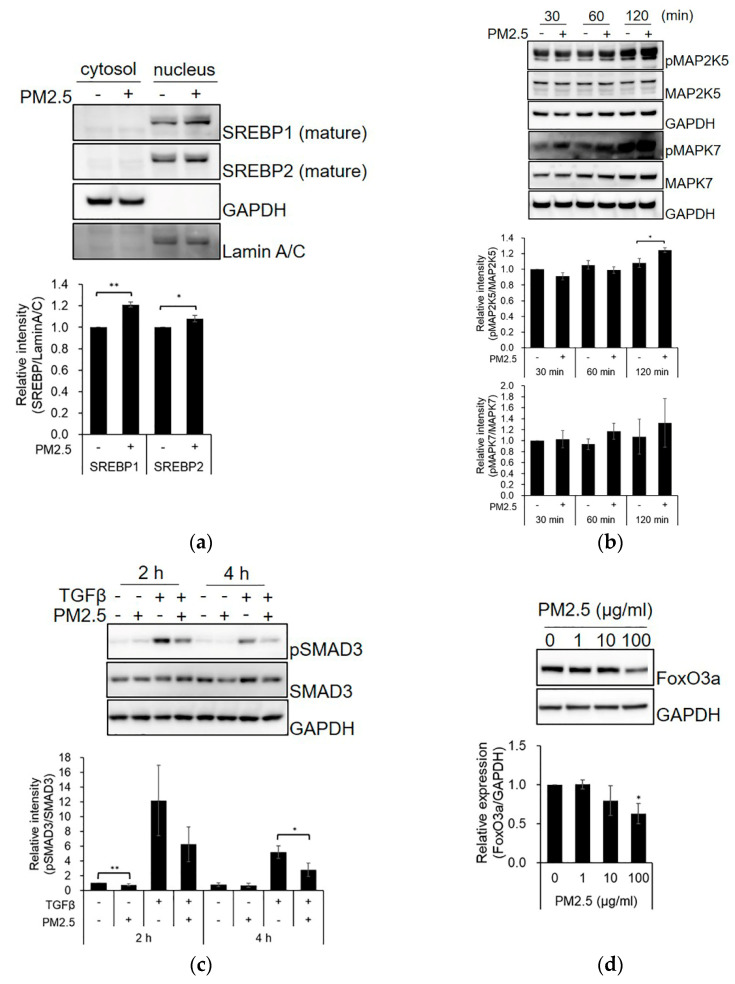
PM_2.5_ affects SREBP-, MAPK-, TGFβ-SMAD3-, and FoxO3a-related signaling. (**a**) SZ95 sebocytes were treated with PM_2.5_ (100 μg/mL) for 48 h. The cells were lysed with hypotonic buffer, and the cytosolic fraction and nuclei were separated. SZ95 sebocytes were treated with PM_2.5_ or TGFβ for the indicated times (**b**,**c**) or 48 h (**d**). The expression levels were analyzed by SDS-PAGE and immunoblotting for the indicated antibodies. The relative intensity of proteins was quantified using the Image J program. Data were analyzed by Student’s *t*-test (* *p* < 0.05, ** *p* < 0.01 vs. untreated control; n = 3).

We validated the signaling pathways of the predicted upstream regulators. The processed mature forms of SREBP1 and SREBP2 were found to be higher in the nucleus of PM_2.5_-treated SZ95 sebocytes than those in the control group (Figure 2a and Figure S4a). Moreover, PM_2.5_ treatment increased the phosphorylation of MAP2K5 and MAPK7 (Figure 2b and Figure S4b) and inhibited TGFβ-induced phosphorylation of SMAD3 compared with the control (Figure 2c and Figure S4c). The expression level of FoxO3a was decreased by PM_2.5_ (Figure 2d and Figure S4d). These results indicate that alterations in SREBP-, MAPK-, TGFβ-SMAD-, and FoxO3a-signaling activity by PM_2.5_ exposure may contribute to the induction of alterations in SZ95 sebocytes.

### 2.4. Effect of PM_2.5_ on Lipid Production, ROS Generation, and Lipid-Peroxidation in SZ95 Sebocytes

**Figure 3:**
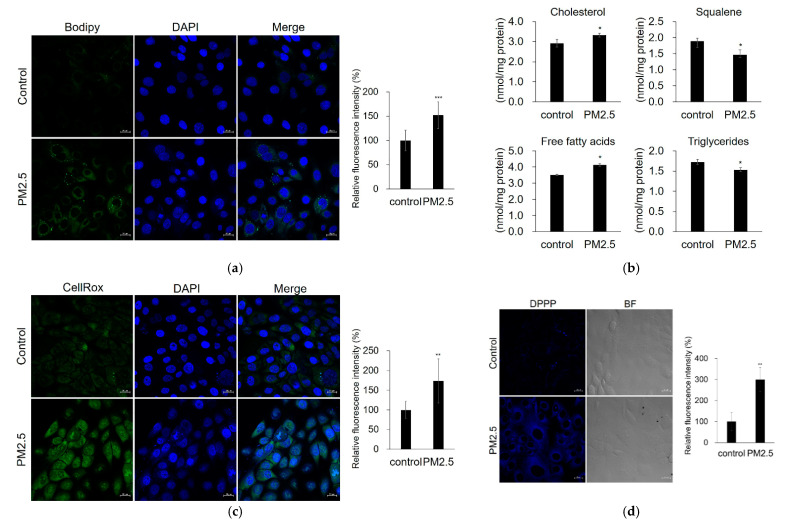
PM_2.5_ increases lipid production, ROS generation, and lipid peroxidation. SZ95 sebocytes were treated with PM_2.5_ (100 μg/mL). (**a**) Lipid production was analyzed by Bodipy 493/503 staining (green) and confocal microscopy. (**b**) Relative levels of major lipid classes were determined by LC–MS/MS. (**c**) ROS generation was analyzed by CellROX Green. (**d**) Lipid peroxidation was measured by DPPP staining (blue). Nuclei were stained with DAPI (blue). The fluorescence intensity was calculated using the Zen software and normalized to the DAPI fluorescence intensity (lipid production and ROS generation) or cell number (lipid peroxidation). Data were analyzed by Student’s *t*-test (* *p* < 0.05, ** *p* < 0.01, *** *p* < 0.001 vs. control). Original magnification: 400×. Scale bars: 20 µm.

According to the lipid staining results, lipid content was increased by PM_2.5_ (Figure 3a). Furthermore, using liquid chromatography–tandem mass spectrometry (LC-MS/MS), we found that PM_2.5_ induced lipid compositional changes, with increased levels of cholesterol and free fatty acids (FAs) and decreased levels of squalene and triglycerides compared with the control (Figure 3b). We also tested whether PM_2.5_ increases ROS generation and lipid peroxidation. We found that PM_2.5_ increased intracellular ROS generation and lipid peroxidation (Figure 3c,d). These results indicate that PM_2.5_ can cause damage, including increased lipid synthesis, ROS generation, lipid peroxidation, and altered lipid composition in sebocytes.

### 2.5. Confirmation of PM_2.5_ Effects on Human Skin Tissue

**Figure 4:**
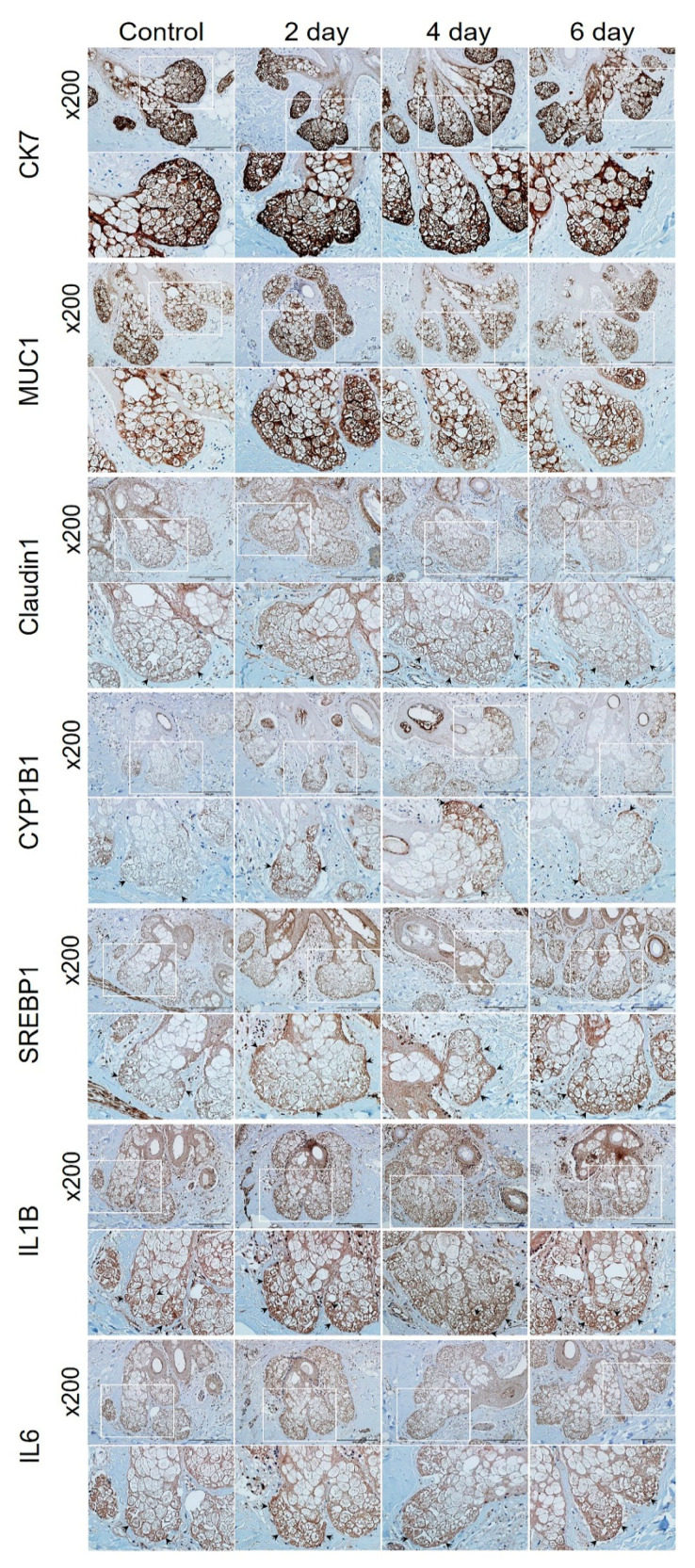
Immunohistochemical analysis of HairSkin^®^. HairSkin^®^ was treated with PM_2.5_ (20 µg/ cm^2^) for the indicated number of days. HairSkin^®^ sections were stained with each antibody of cytokeratin 7 (CK7), mucin 1 (MUC1), claudin1, CYP1B1, SREBP1, IL1B, and IL6. Squares marked in white represent the enlarged areas; the arrows point to representative stained regions. Original magnification: 200×. Scale bars: 200 µm.

Immunohistochemical analysis was performed on human tissue (HairSkin^®^) to confirm the PM_2.5_-induced alterations. The expression levels of CYP1B1, SERBP1, IL1B, and interleukin 6 (IL6) were increased, and the expression of claudin1 was decreased by PM_2.5_ (Figure 4). Cytokeratin 7 and mucin 1 were used as markers for the sebaceous glands.

## 3. Discussion

In this study, we demonstrated the harmful effects of PM_2.5_ on SZ95 sebocytes and human skin tissue through transcriptome analysis. PAHs, which are components of PM_2.5_, regulate the expression of xenobiotic-metabolizing enzymes (XMEs) through the AHR/AHR nuclear translocator (ARNT) [21]. XMEs are classified into phase I, adding functional groups such as an oxide and epoxide into the substrate, and phase II, conjugating with moieties such as glucuronide, acetate, sulfate, glutamine, thiocyanate, and glucoside to facilitate excretion [28]. In our transcriptome analysis, the expression of type I XMEs, such as CYP450 (CYP1A1 and CYP1B1), aldehyde dehydrogenase (ALDH1L1, ALDH3A1, and ALDH1A1), and prostaglandin-endoperoxide synthases (PTGS1), and type 2 XMEs, such as UDP glucuronosyltransferases (UGT1A6 and UGT1A3), glutathione S-transferases, mu 2 (GSTM2), and sulfotransferases (SULT1A2 and SULT1E1), were highly altered by PM_2.5_.

The expression of ALDH1A1, a well-known enzyme for all-trans-retinoic acid (ATRA), was downregulated by PM_2.5_. We also found that the expression of retinol dehydrogenase 10 (RDH10), an enzyme that oxidizes retinol into retinal aldehyde, and retinoic acid receptor beta (RARB), an ATRA receptor, was decreased by PM_2.5_ (Figure 1b) [22,29]. As ATRA reduces lipid synthesis in the sebaceous gland, a low concentration of ATRA is important for the function of sebaceous glands that produce lipids. Additionally, we found that the expression of hydroxysteroid 17-beta dehydrogenase 1 (HSD17B1), which reduces estrone to 17β-estradiol (the most active form), was downregulated in PM_2.5_-treated SZ95 sebocytes (Figure 1b) [30]. Furthermore, CYP1A1 was capable of catalyzing the 2-hydroxylation of estradiol to produce less active estrogen [31]. Estrogen (17β-estradiol) inhibited lipogenesis in the sebaceous glands [8]. Thus, these results indicate the possibility that PM_2.5_ causes the downregulation of ATRA-mediated signaling and the reduction of active estrogen; the altered retinol metabolism and steroid hormone metabolism subsequently affect sebocyte functions, such as lipid synthesis.

There are differences in the sebum of subjects in areas with and without severe pollution. Increased sebum excretion rates and lower concentrations of squalene were observed in highly polluted regions [32,33]. Liao et al. reported that PM_2.5_ disrupts barrier functions by upregulating cholesterol synthesis in human keratinocytes and a three-dimensional epidermis tissue model [34]. Kwack et al. showed that PM_10_ (100 μg/mL) increases the expression of inflammatory cytokines and ROS production in sebocytes and upregulates sebum production and inflammatory nodule production in *Cutibacterium acnes*-induced mice [35]. The expression of various genes related to lipid metabolism changed in our RNA-seq analysis, and the activation of signaling pathways related to cholesterol biosynthesis was analyzed using IPA. As a result, PM_2.5_ increased lipid synthesis and peroxidation. Moreover, it altered the lipid composition by decreasing squalene and triglyceride levels and increasing cholesterol and free FA levels. In addition, the expression of stearoyl-CoA desaturase (SCD), an FA desaturase enzyme, was elevated in PM_2.5_-treated SZ95 sebocytes (Figure 1b,c). Increased sebum production, alterations in lipid composition, increased sebum oxidation particularly for squalene, and the altered ratio between saturated and unsaturated FAs in skin diseases such as acne have been reported [36,37,38,39]. Triglycerides are catalyzed to free FAs by resident and microflora lipases, such as *Propionibacterium acnes*, leading to the activation of inflammasome [40]. Moreover, free FAs, such as palmitic acid, induce lipid synthesis and increase the secretion of IL6 and IL8 [41]. Therefore, alterations in lipid metabolism and the increase in lipid peroxidation by PM_2.5_ may result in the dysfunction of sebaceous glands and the development of skin diseases. In our analysis, SREBP-, TGFβ-SMAD-, MAPK-, and FoxO3a-related signaling pathways were predicted with PM_2.5_-induced phenomena-related signaling pathways. SREBPs regulate lipid homeostasis by activating the genes required for lipid synthesis, including cholesterol, FAs, and triacylglycerol as transcription factors [24]. Furthermore, PM induces inflammasomes by activating SREBP1 in human pulmonary fibroblasts [23]. TGFβ signaling maintains sebocytes in an undifferentiated state by decreasing the expression of lipogenesis-related genes [27]. MAPKs regulate a wide range of cellular homeostatic processes, including proliferation, differentiation, survival, apoptosis, and responses to stress conditions. Suppression of MAP2K5–MAPK7 signaling downregulates SREBP target genes and decreases lipid synthesis in small cell lung cancer cells [25]. The transcription factor FoxO3 regulates biological processes such as apoptosis, cell cycle, oxidative stress resistance, and inflammation [42]. Melnik reported that ATRA promoted apoptosis by upregulating the expression of FoxO3a and its direct target, tumor necrosis factor (ligand) superfamily member 10 (TNFSF10), thereby downregulating sebum secretion [26,43]. In our results, PM_2.5_ decreased the expression of TNFSF10 and FoxO3a (Figure 1b and Figure 2d). Although the changes in activity were not validated in this study, wnt-β-catenin signaling pathway-related factors, such as catenin beta 1 (CTNNB1), WNT3A, and low-density lipoprotein receptor-related protein 6 (LRP6), were predicted by IPA to be inhibited upstream regulators (Table 2). The wnt-β-catenin signaling pathway plays a role in regulating the differentiation of sebaceous glands [12,44,45]. Thus, wnt-β-catenin signaling may be associated with PM_2.5_-induced sebocyte damage.

In summary, this study investigated the effects of PM_2.5_ on SZ95 sebocytes via transcriptome analysis. We showed that PM_2.5_ increased lipid synthesis, lipid peroxidation, inflammatory cytokine expression, and oxidative stress; moreover, it altered lipid composition and expression of cell barrier-related proteins in SZ95 sebocytes. We also identified the signaling pathways related to the harmful effects of PM_2.5_ and validated the altered activity of the signaling pathways in PM_2.5_-exposed sebocytes and human skin models. Further studies are required to elucidate the causal relationship between skin irritant reaction by PM_2.5_ through hair follicles and skin disease aggravations. Nevertheless, the elucidated molecular alterations in the expression levels of DEGs and analysis of the associated mechanisms provide novel biomarkers and therapeutic targets to relieve the symptoms of skin damage caused by PM_2.5_.

## 4. Materials and Methods

### 4.1. PM_2.5_ Preparation and Analysis

Ambient PM_2.5_ was collected on a polytetrafluoroethylene filter (Zefluor™; Pall Life Science, Mexico City, Mexico) using a high-volume sampler machine (TE6070; Tisch Environmental Inc., Cleves, OH, USA) equipped with a PM_2.5_ selective-inlet head at a flow rate of 1.13 m^3^/min. Sample collection of PM2.5 was carried out on the rooftop of the Amorepacific Corporation R&I building located in Yongin, Korea (37°15′ N, 127°06′ E), from January to April 2018. PM_2.5_ was extracted from the collected samples and resuspended in 20% ethanol. The detailed methods were described in a previous study [3,7].

### 4.2. Cell Culture

The immortalized human sebaceous gland cell line SZ95 (SZ95 sebocyte), which has been demonstrated to express the characteristics of human sebocytes [19], was cultured in Dulbecco’s modified Eagle medium (DMEM)/F-12 containing 10% heat-inactivated fetal bovine serum, 1% penicillin/streptomycin (P/S; Gibco, Grand Island, NY, USA), 5 ng/mL human epidermal growth factor, and 1 mM CaCl_2_ (Sigma-Aldrich, St. Louis, MO, USA). Cells were maintained in a humidified atmosphere of 5% CO_2_ at 37 °C; the medium was replaced every 2–3 days. SZ95 sebocytes were used at 30–39 passages and 80–90% confluence for the experiments. SZ95 sebocytes were treated with 20% ethanol or PM_2.5_ (100 μg/mL) in phenol-red free DMEM/F-12 (Gibco, Grand Island, NY, USA) containing 1% P/S for 24 h for RNA-seq.

### 4.3. RT-qPCR Analysis

Total RNA was isolated using TRIzol reagent (Ambion, Carlsbad, CA, USA) according to the manufacturer’s protocol and quantified using a NanoDrop spectrophotometer (Thermo Scientific, Waltham, MA, USA). cDNA was generated from 1 μg of RNA using the RevertAid First Strand cDNA Synthesis Kit (Invitrogen, Carlsbad, CA, USA) according to the manufacturer’s protocol. RT-qPCR was performed in a solution containing 1 μL of cDNA, TaqMan Universal Master Mix II (Applied Biosystems, Foster City, CA, USA), and TaqMan probes using a 7500 Fast Real-Time PCR system (Applied Biosystems, Foster City, CA, USA). The parameters and TaqMan probes that were used are summarized in the Supplementary Material.

### 4.4. Western Blotting of Cell Lysates and ELISA

Sub-confluent cells were treated with PM_2.5_ or TGFβ (5 ng/mL) for various durations. To measure the IL6 secretion level, the medium was spun down at 2000 rpm for 5 min and analyzed using an ELISA kit (#BMS213HS; Invitrogen, Vienna, Austria) according to the manufacturer’s protocol. To detect nuclear proteins, fractionation of cytosolic and nuclear proteins was performed using a hypotonic buffer. For Western blot analysis, cell lysates were resuspended in a sodium dodecyl sulfate (SDS) sample buffer containing a reducing buffer, resolved by SDS-polyacrylamide gel electrophoresis (PAGE), and transferred to a polyvinylidene difluoride membrane. The antibodies that were used are listed in the Supplementary Material.

### 4.5. Measurement of Lipid Production

SZ95 sebocytes were seeded on glass coverslips coated with collagen I and were then treated with PM_2.5_ (100 μg/mL) for 48 h. Following incubation, the cells were washed with phosphate-buffed saline (PBS) and treated with Bodipy 493/503 (2 μM; Invitrogen, Eugene, OR, USA) for 30 min at 37 °C. Subsequently, the cells were washed thrice with PBS and fixed with 4% paraformaldehyde. A mounting medium containing DAPI (P36962; Invitrogen, Eugene, OR, USA) was used for nuclear staining. The cells were observed under a confocal microscope (LSM980; Zeiss, Oberkochen, Germany).

### 4.6. Measurement of ROS and Lipid Peroxidation

SZ95 sebocytes were seeded on glass coverslips coated with collagen I. For ROS measurements, the cells were treated with PM_2.5_ for 2 h and with CellROX Green (5 μM; Life Technologies, Carlsbad, CA, USA) for the last 30 min. The cells were then washed thrice with PBS and fixed with 4% paraformaldehyde. A mounting medium containing DAPI was used for nuclear staining. For detection of lipid peroxidation, the SZ95 sebocytes were treated with PM_2.5_ for 24 h and then 5 μM diphenyl-1-pyrenylphosphine (DPPP; Invitrogen, Eugene, OR, USA) for 30 min at 37 °C. The cells were observed under a confocal microscope (LSM980; Zeiss, Oberkochen, Germany).

### 4.7. Human Skin Tissue Model and Immunohistochemistry

HairSkin^®^ models purchased from Genoskin (Toulouse, France) were cultured in a humidified atmosphere of 5% CO_2_ at 37 °C for 24 h, and PM_2.5_ (20 μg/cm^2^) was applied on the surface of the HairSkin^®^ models for 0, 2, 4, or 6 days. HairSkin^®^ models were fixed with 10% formalin (Sigma-Aldrich, St. Louis, MO, USA) for paraffin-embedded sectioning. The paraffin-embedded tissues were sliced into 3-μm sections and stained with hematoxylin and eosin, along with the specific antibodies listed in the supplementary materials. The stained sections were visualized under an optical microscope (Olympus, Tokyo, Japan).

## Figures and Tables

**Table 1:** Top canonical pathways of DEGs identified by Ingenuity Pathway Analysis (IPA). Top canonical pathways were analyzed using DEGs (|fold changes| > 1.5, *p*-value < 0.01). Z-score indicates the predicted activation (positive value) or inhibition (negative value) of the canonical pathway. Ratio is calculated by dividing the number of the DEGs in a pathway by the total number of genes that make up the pathway. Molecules are genes in the DEGs that are related to the pathway. *p*-values were calculated by right-tailed Fisher’s exact tests.

Ingenuity Canonical Pathways	−Log (*p*-Value)	Ratio	*z*-Score	Molecules
Xenobiotic metabolism AHR signaling pathway	6.41	0.23	3.578	ABCG2, AHRR, ALDH1A1, ALDH1L1, ALDH3A1, ALDH3B1, ALDH3B2, ALDH6A1, CYP1A1, CYP1B1, GSTA4, GSTM2, GSTM3, GSTM4, IL1A, IL1B, IL6, UGT1A1, UGT1A3, UGT1A6
Superpathway of cholesterol biosynthesis	5.17	0.345	3.162	ACAT2, CYP51A1, DHCR7, FDFT1, FDPS, HMGCR, HMGCS1, LSS, MSMO1, MVD
Cholesterol Biosynthesis I	3.07	0.385	2.236	CYP51A1, DHCR7, FDFT1, LSS, MSMO1
Cholesterol Biosynthesis II (via 24,25-dihydrolanosterol)	3.07	0.385	2.236	CYP51A1, DHCR7, FDFT1, LSS, MSMO1
Cholesterol Biosynthesis III (Via Desmosterol)	3.07	0.385	2.236	CYP51A1, DHCR7, FDFT1, LSS, MSMO1
Role of IL-17A in psoriasis	2.9	0.357	−2.236	CXCL1, CXCL6, CXCL8, S100A8, S100A9
Thyroid cancer signaling	2.4	0.152	−2.887	CCND1, CXCL8, FOS, IRS1, jun, myc, PIK3R3, RAP2B, RASD2, TCF4, TCF7L1, TP53
Superpathway of Geranylgeranyldiphosphate Biosynthesis I (via mevalonate)	2.36	0.278	2.236	ACAT2, FDPS, HMGCR, HMGCS1, MVD
Estrogen-dependent breast cancer signaling	1.96	0.139	−2.236	AKR1C1/AKR1C2, CCND1, FOS, HSD17B1, HSD17B14, HSD17B2, HSD17B3, JUN, PIK3R3, RAP2B, RASD2
eNOS signaling	1.09	0.0943	2.496	BDKRB1, CALML5, CAV1, CCNA1, CHRNB4, ESR2, GUCY1B1, HSPA5, KDR, LPAR1, LPAR3, PGF, PIK3R3, PRKAA2, PRKD1

**Table 2:** Upstream regulators of DEGs. The top upstream regulators of DEGs (|fold changes| > 1.5, *p*-value < 0.01) in PM_2.5_ -treated SZ95 sebocytes were analyzed with IPA. Activation z-score indicates the predicted activation (positive value) or inhibition (negative value) of the upstream regulator. The *p*-value of overlap indicates the statistical significance of the overlap of the DEGs and genes regulated by an upstream regulator. *p*-values were calculated by right-tailed Fisher’s exact tests.

Upstream Regulator	Molecule Type	Predicted Activation State	Activation *z*-Score	*p*-Value of Overlap
SREBF1	Transcription regulator	Activated	4.149	1.11 × 10^−14^
SREBF2	Transcription regulator	Activated	4.063	1.41 × 10^−13^
MAPK7	Kinase	Activated	2.935	1.49 × 10^−11^
SCAP	Other	Activated	4.12	3.39 × 10^−10^
MAP2K5	Kinase	Activated	3.704	1.59 × 10^−9^
DSCAML1	Other	Activated	2.294	1.41 × 10^−8^
EWSR1-FLI1	Fusion gene/product	Activated	2.426	1.72 × 10^−8^
CYP7A1	Enzyme	Activated	2.333	3.62 × 10^−7^
DSCAM	Other	Activated	3.286	8.32 × 10^−7^
SH3TC2	Other	Activated	2.111	1.2 × 10^−6^
CTNNB1	Transcription regulator	Inhibited	−2.587	4.66 × 10^−16^
SMAD3	Transcription regulator	Inhibited	−2.105	6.62 × 10^−14^
WNT3A	Cytokine	Inhibited	−3.324	1.21 × 10^−12^
TGFB1	Growth factor	Inhibited	−2.179	3.34 × 10^−12^
INSIG1	Other	Inhibited	−3.761	4.37 × 10^−11^
LRP6	Transcription regulator	Inhibited	−2.619	1.58 × 10^−9^
MRTFB	Transcription regulator	Inhibited	−2.939	8.37 × 10^−9^
FOXO3	Transcription regulator	Inhibited	−3.051	1.71 × 10^−8^
PDGF BB	Complex	Inhibited	−2.768	3.16 × 10^−8^
MFSD2A	Transporter	Inhibited	−3.293	3.76 × 10^−8^

**Table 3:** Top disease and biologic functions analyzed with IPA (positive *z*-score value). Disease and biologic functions of DEGs (|fold changes| > 1.5, *p*-value < 0.01) in PM_2.5_-treated SZ95 sebocytes were analyzed with IPA. Z-score indicates the predicted activation (positive value) or inhibition (negative value) of the disease or function. Bias-corrected z-score is a statistically corrected value to avoid false predictions. No. of molecules is the number of genes in DEGs associated with each function. *p*-values were calculated by right-tailed Fisher’s exact tests and Benjamini–Hochberg correction (B–H *p*-value).

Categories	Diseases or Functions Annotation	*p*-Value	B–H *p*-Value	Predicted Activation State	Activation z-Score	Bias-Corrected z-Score	No. of Molecules
Lipid metabolism, molecular transport, small molecule biochemistry	Concentration of lipid	1.42 × 10^−14^	1.79 × 10^−12^	Increased	2.871	2.767	152
dermatological diseases and conditions, organismal injury and abnormalities	Abnormality of skin morphology	3.81 × 10^−8^	1.66 × 10^−6^	Increased	2.646	2.853	69
lipid metabolism, small molecule biochemistry	Fatty acid metabolism	6.95 × 10^−15^	9.14 × 10^−13^	Increased	2.611	2.078	111
cancer, organismal injury and abnormalities, respiratory disease	Development of lung tumor	3.6 × 10^−10^	2.3 × 10^−8^	Increased	2.566	2.766	273
organismal survival	Organismal death	1.08 × 10^−17^	1.78 × 10^−15^	Increased	2.547	4.19	380

## Data Availability

All NGS data files are available from the National Center for Biotechnology Information Gene Expression Omnibus (NCBI GEO, https://www.ncbi.nlm.nih.gov/geo/), accession number GSE213620.

## Supplementary Materials

The supporting information can be downloaded at: https://www.mdpi.com/article/10.3390/ijms231911534/s1. References [46,47,48,49,50,51,52,53,54] are cited in the supplementary materials.
