# Supplementary material for: Transcriptome Analysis of Particulate Matter 2.5-Induced Abnormal Effects on Human Sebocytes
Source: Int J Mol Sci. 2022 Sep 29;23(19):11534. doi: 10.3390/ijms231911534 (PMC9570376; doi:10.3390/ijms231911534)
Supplement: Supplementary file 1 [file ijms-23-11534-s001.zip › ijms-1864572-supplementary.pdf]

Supplementary Materials

Transcriptome analysis of particulate matter 2.5-induced abnormal effects on human sebocytes

Hye-Won Na[1], Hyun Soo Kim[2], Hyunjung Choi[1], Nari Cha[1], Young Rok Seo[2], Yong Deog Hong[1], Hyoung-June Kim[1*]

[1] Research and Innovation Center, AMOREPACIFIC, Yongin 17074, Gyeonggi-do, Korea

[2] Department of Life Science, Institute of Environmental Medicine, Dongguk University Biomedi Campus, Goyang 10326, Korea

[*] Correspondence: leojune@amorepacific.com; Tel.: 82-31-280-5827; Fax: 82-31-899-2595

Supplementary materials and methods

Cell viability test

Cell viability was assessed using the Cell Counting Kit-8 (Dojindo Laboratories, Kumamoto, Japan) according to the manufacturer's protocol.

RNA-sequencing (RNA-seq) and differential expression analysis

SZ95 sebocytes treated with 20% ethanol or $PM_{2.5}$ were used in RNA-sequencing. Total RNA was isolated using Trizol (QIAzol Lysis Reagent; Qiagen, Hilden, Germany) and qualified using a 2100 Bioanalyzer (Agilent, Santa Clara, CA, USA.). cDNA libraries were generated using the TruSeq Stranded mRNA LT Sample Prep Kit (Illumina, San Diego, CA, USA) according to the manufacturer's guidelines. All samples were sequenced with 101 bp paired-end reads using Illumina HiSeq 2500 according to the user guide. The libraries were quantified using quantitative real-time PCR (qPCR) according to the Illumina qPCR Quantification Protocol Guide and Roche Rapid Library Standard Quantification solution and calculator. An Agilent Technologies 2100 Bioanalyzer using a DNA 1000 chip was used to determine the template size distribution. To remove adapter contamination, reads were trimmed using Trimmomatic 0.32 [46]. The trimmed reads were mapped to the human genome (UCSC hg19) using HISAT2 (version 2.0.5) [47] and assembled using StringTie (version1.3.3b) [48,49]. The abundance and gene levels were determined based on the

fragments per kilobase of transcript per million fragments mapped. Analysis of differently expressed genes (DEGs) was performed using DESeq2. Raw *p*-values from differential expression analyses were determined using the negative binomial Wald test, and adjusted *p*-values were determined using the Benjamini and Hochberg test.

Gene set enrichment analysis and Ingenuity Pathway Analysis (IPA)

Gene set enrichment analysis of DEGs was performed using the Gene Ontology (GO, http://geneontology.org/) and Kyoto Encyclopedia of Genes and Genomes (KEGG, http://www.genome.jp/kegg/) databases. Subsequently, IPA (Qiagen) was used to predict the canonical pathways, upstream regulators, and downstream effects related to disease and biological functions [50].

Real-time qPCR analysis

The parameters were as follows: initial denaturation (10 min), 40 cycles of amplification at 95 °C (15 s), and annealing and extension at 60 °C (60 s). The data were analyzed using the $2^{-\Delta\Delta Ct}$ method. The following TaqMan probes (Applied Biosystems) were used: cytochrome P450 family 1 subfamily A member 1 (CYP1A1; Hs01054797_g1), CYP1B1 (Hs00164383_m1), aryl-hydrocarbon receptor repressor (AHRR; Hs01005075_m1), metallothionein 1E (MT1E; Hs01938284_g1), CYP3A7 (Hs02511627_s1), sulfotransferase family 1E member 1 (SULT1E1; Hs00960938_m1),

aldehyde dehydrogenase 1 family member A1 (ALDH1A1; Hs00946916_m1), interleukin 1 beta (IL1B; Hs01555410_m1), IL6 (Hs00174131_m1), tumor necrosis factor superfamily member 10 (TNFSF10; Hs00921974_m1), klotho (KL; Hs00934627_m1), prostaglandin-endoperoxide synthase 1 (PTGS1; Hs00377726_m1), acyl-CoA oxidase like (ACOXL; Hs01102524_m1), stearoyl-CoA desaturase (SCD; Hs01682761_m1), insulin induced gene 1 (INSIG1; Hs00356479_g1), 3-hydroxy-3-methylglutaryl-CoA synthase 1 (HMGCS1; Hs00940429_m1), insulin receptor substrate 1 (IRS1; Hs00178563_m1), claudin11 (Hs00194440_m1), claudin1 (Hs00221623_m1), retinol dehydrogenase 10 (RDH10; Hs00416907_m1), retinoic acid receptor beta (RARB; Hs00977140_m1), hydroxysteroid 17-beta dehydrogenase 1 (HSD17B1; Hs00166219_g1), and ribosomal protein L13a (RPL13A; Hs04194366_g1).

Antibodies for western blotting of cell lysates

The following antibodies were used: CYP1A1 (sc-25304) and sterol regulatory element binding transcription factor 1 (SREBP1; sc-13551) from Santa Cruz (Paso Robles, CA, USA) and CYP1B1 (ab185954); IL1B (ab9722), SCD (ab19862), and HMGCS1 (ab155787) from Abcam (Waltham, MA, USA); Claudin1 (H00009076-M01; novus, Centennial, CO, USA); and SREBP2 (MAB7119; R&D Systems, Minneapolis, MN, USA). Mitogen-activated protein kinase (MAPK) kinase 5 (MAP2K5; PA5-29236) and pMAP2K5 (PA5-37701) were from Invitrogen. MAPK7 (#3372), pMAPK7 (#3371), pSMAD3 (#9520), SMAD3 (#9523), Forkhead Box O3 (FoxO3a; #12829) and GAPDH

(#2118) were from Cell Signaling Technology (Beverly, MA, USA). Detection was performed using horseradish peroxidase-conjugated anti-mouse IgG or goat anti-rabbit secondary antibodies (Cell Signaling Technology). Detection was performed using a chemiluminescence reagent (SuperSignal West; Thermo Fisher Scientific, Cleveland, OH, USA).

Measurement of lipid contents

Quantification of lipids containing squalene, triglycerides, and free fatty acids (FA) was performed using 20% ethanol- or $PM_{2.5}$-treated SZ95 sebocytes. To measure the levels of lipids containing squalene, triglycerides, and free fatty acids (FA), lipid extraction was performed using the Folch method with minor modifications [51]. Briefly, RIPA lysis buffer with a complete protease inhibitor cocktail together with harvested cell pellets were homogenized. Protein levels were determined using a Thermo Protein Assay Kit (Pierce, Rockford, IL, USA) according to the manufacturer's instructions.

Quantification of squalene lipid extraction was performed as previously reported [52], with minor modifications. The squalene level (expressed as nmol/mg protein) was quantified using an HPLC system equipped with a photodiode array detector at a wavelength of 205 nm (Agilent 1260 HPLC system; Agilent).

To measure triglyceride, free cholesterol, and free FA levels, the cell lysates were

lysed and sonicated in methanol–chloroform (1:2, v/v) containing butylated hydroxytoluene (500 μg/mL), followed by the addition of 500 pmol docosahexaenoic acid, cholesterol-d6, and 1,3-heptadecanoin-2-heptadecanoin glyceride (d5-TG 51:1) as an internal standard. The extracted lipids were dried in a vacuum system, re-dissolved in methanol, and analyzed using liquid chromatography–electrospray ionization–tandem mass spectrometry (LC–ESI–MS/MS; API 5500 QTrap mass; Applied Biosystems/MDS Sciex, Toronto, Canada) in the selective ion monitoring mode. Free cholesterol and FAs were separated using reverse-phase HPLC (ExionLC™ Series UHPLC, Applied Biosystems, Waltham, MA, USA) on a Kinetex C18 column (2.1 × 100 mm, internal diameter: 2.6 μm; Phenomenex, St. Louis, MO, USA), as previously reported [53,54]. All data were acquired using the Analyst 1.7.1 software (Applied Biosystems).

Human skin tissue model and immunohistochemistry

Human skin explant HairSkin® models purchased from Genoskin (Toulouse, France) were collected from the skin biopsies of healthy donors. Genoskin obtained the samples with the informed consent of the individual donors, and the procedure was approved and authorized by the French Ethics Committee (Comité de Protection de Personnes) and the French Ministry of Research (AC-2017-2897, 12 Oct 2017). The sliced paraffin-embedded tissues were stained with the following antibodies; cytokeratin 7 (CK7; MA1-90894; Invitrogen), claudin1 (H00009076-M01; novus), and

SREBP1 (NB100-2215; novus). Mucin 1 (MUC1; ab15481), CYP1B1 (ab185954), IL1B (ab9722), and IL6 (ab6672) were purchased from Abcam.

Supplementary Figures

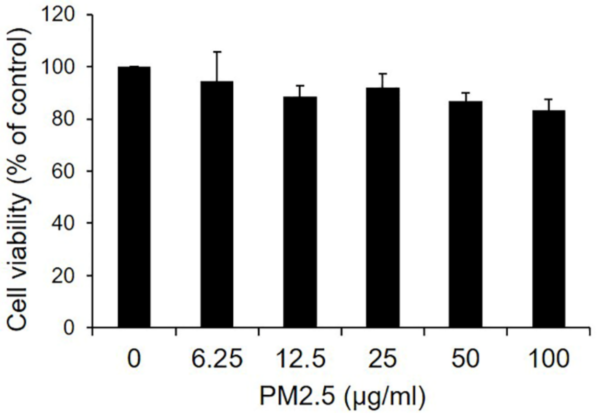

Figure S1. Effect of $PM_{2.5}$ on cell viability. SZ95 sebocytes were treated with different concentrations of $PM_{2.5}$ for 24 h to test cell viability. Cell viability was determined by a CCK-8 assay.

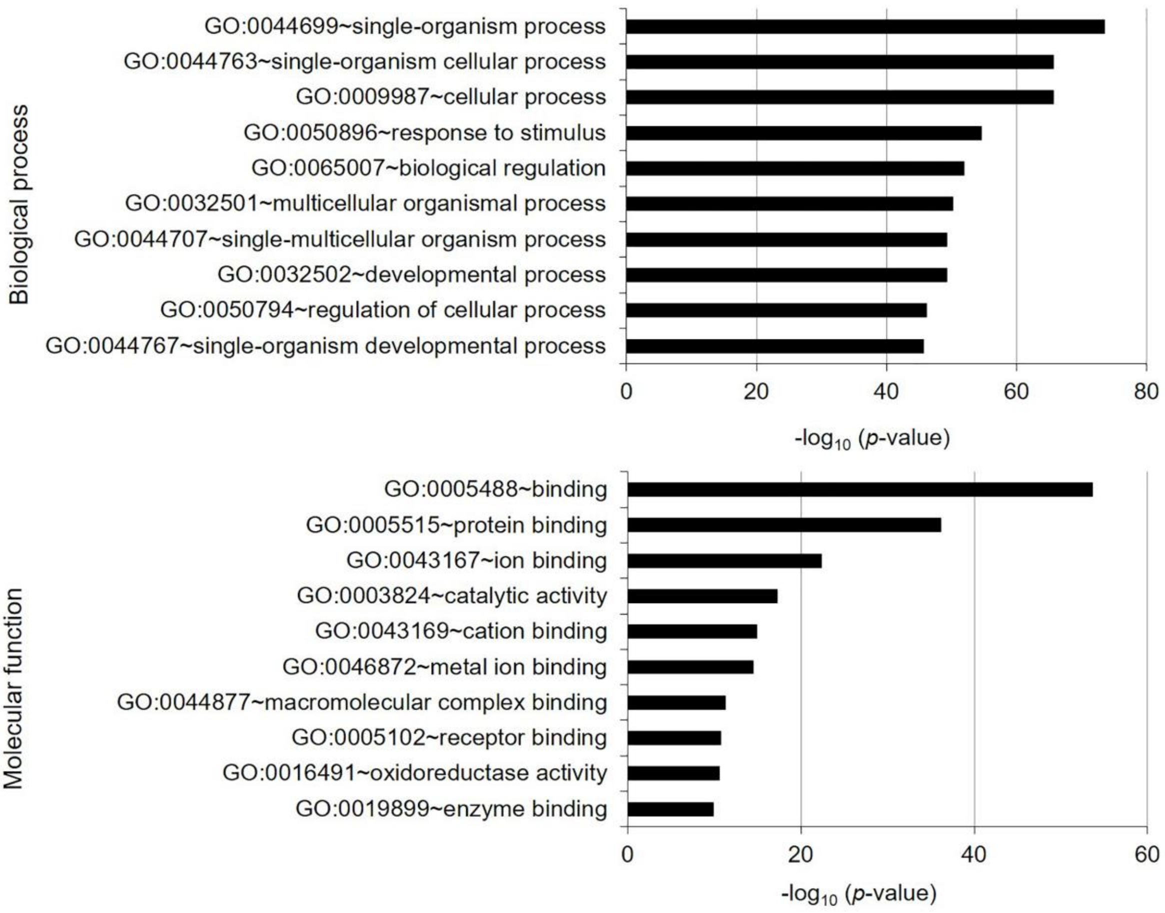

Figure S2. The top ten enriched GO tems. The GO terms were obtained from pathway enrichment analysis of differentially expressed genes (DEGs; |fold changes| > 2, *p*-value < 0.05) in SZ95 sebocytes with $PM_{2.5}$ treatments. Bars represent −log10(p-value). *P*-values were calculated by modified Fisher's exact tests.

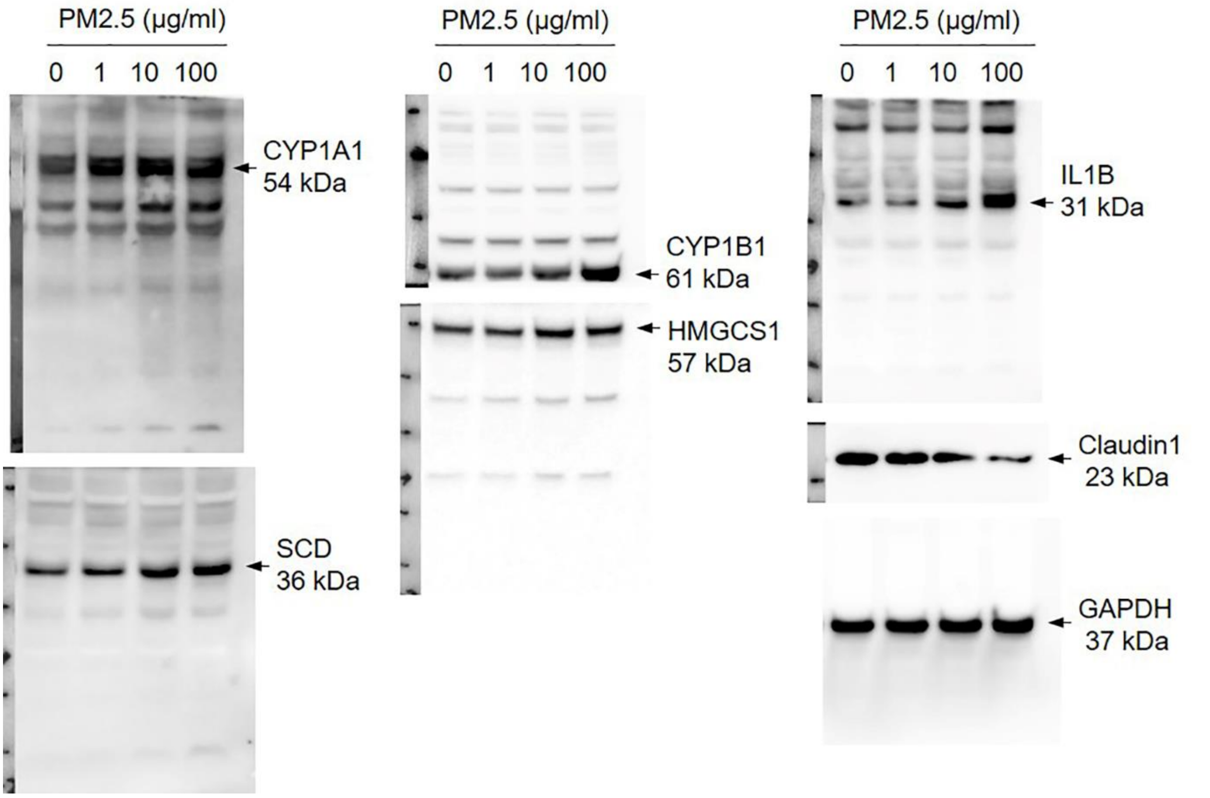

Figure S3. $PM_{2.5}$ alters the expression levels of xenobiotic metabolism, inflammatory response, oxidative stress, lipid metabolism, and cell barrier-related factors. SZ95 sebocytes were treated with $PM_{2.5}$ for 48 h. The cells were lysed with RIPA lysis buffer. The lysate was separated by SDS-PAGE, and immunoblotting was performed. The western blot shows the full blot data for the proteins indicated in Figure 1d, which are cytochrome P450 family 1 subfamily A member 1 (CYP1A1), CYP1B1, stearoyl-CoA desaturase (SCD), 3-hydroxy-3-methylglutaryl-CoA synthase 1 (HMGCS1), claudin 1, interleukin 1 beta (IL1B), and glyceraldehyde 3-phosphate dehydrogenase (GAPDH).

(a)

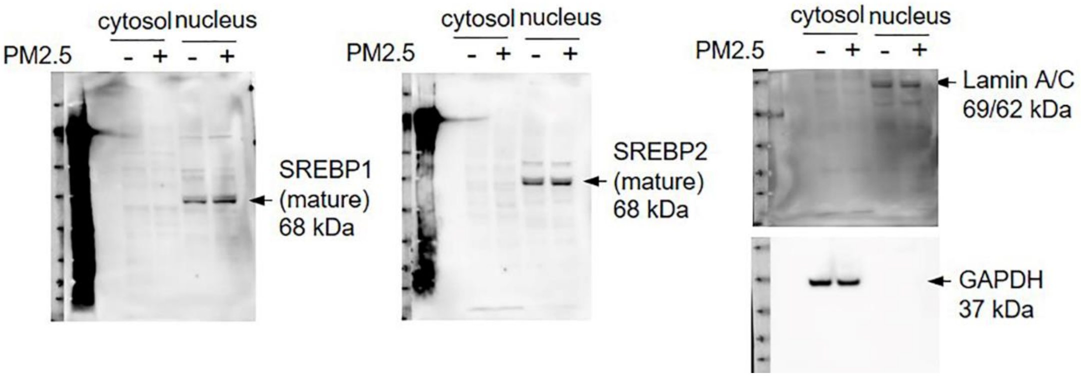

cytosol nucleus
PM2.5 - + - +
SREBP1
(mature)
68 kDa
cytosol nucleus
PM2.5 - + - +
SREBP2
(mature)
68 kDa
cytosol nucleus
PM2.5 - + - +
Lamin A/C
69/62 kDa
GAPDH
37 kDa

(b)

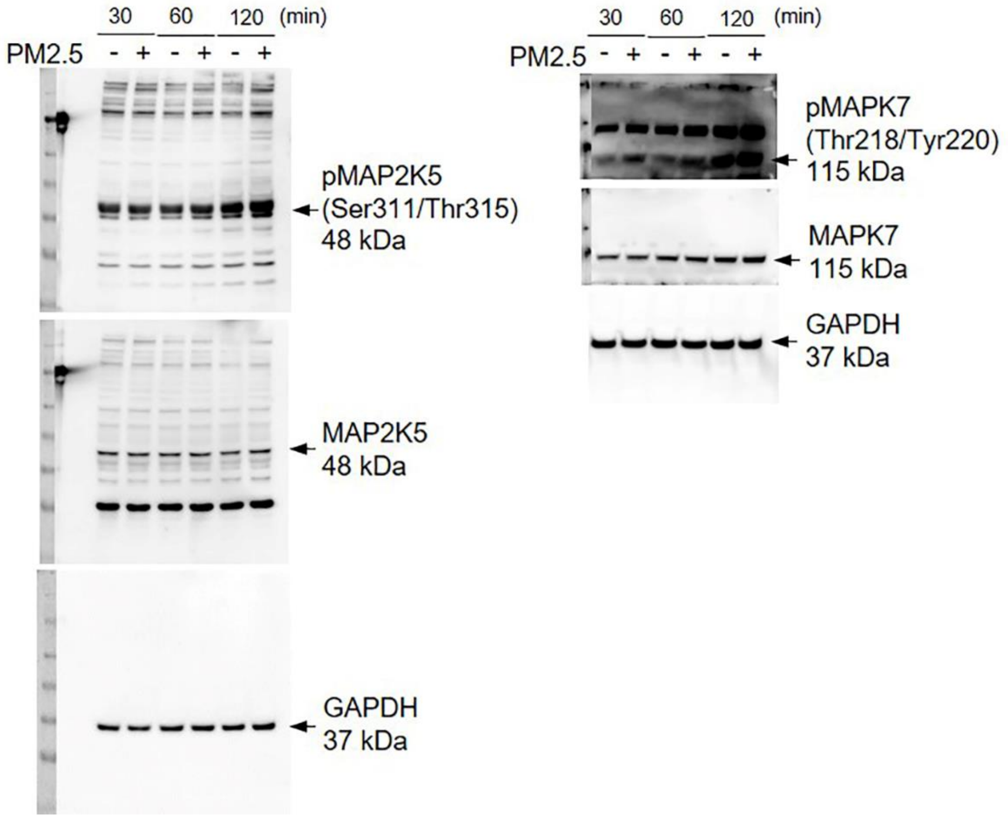

30 60 120 (min)
PM2.5 - + - + - +
pMAP2K5
(Ser311/Thr315)
48 kDa
MAP2K5
48 kDa
GAPDH
37 kDa
30 60 120 (min)
PM2.5 - + - + - +
pMAPK7
(Thr218/Tyr220)
115 kDa
MAPK7
115 kDa
GAPDH
37 kDa

(c)

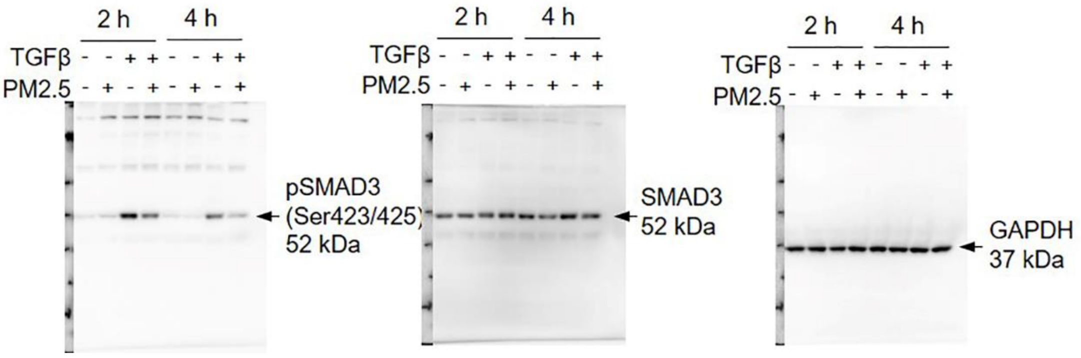

(d)

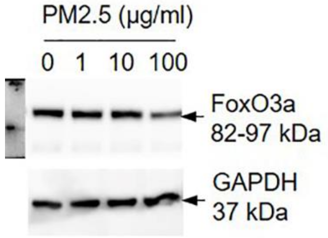

Figure S4. $PM_{2.5}$ affects SREBP-, MAPK-, TGFβ-SMAD3-, and FoxO3a-related signaling. (a) SZ95 sebocytes treated with $PM_{2.5}$ (100 μg/mL) for 48 h. The cells were lysed with hypotonic buffer, then the cytosolic fraction and nuclei were separated. SZ95 sebocytes treated with $PM_{2.5}$ or TGFβ for the indicated times (b,c) or 48 h (d). The cells were lysed with RIPA lysis buffer. The lysate was separated by SDS-PAGE, and immunoblotting was performed for the indicated proteins. The western blot shows the full blot data for the proteins indicated in Figure 2, which are sterol regulatory element binding proteins (SREBPs), lamin A/C, mitogen-activated protein kinase 7 (MAPK7), MAP2K5, SMAD3, forkhead Box O3a (FoxO3a), and GAPDH.

Supplementary Table

Table S1 DEGs (|fold changes| > 2, *p*-value < 0.05) by $PM_{2.5}$ in SZ95 sebocytes. *P*-values were calculated by the negative binomial Wald test and Benjamini–Hochberg correction (adjusted *p*-value).

Gene category	Transcript_ID	Gene Symbol	Description	Fold change	p-value	Adjusted p-value
Xenobiotic metabolism and Detoxification	NM_000499	CYP1A1	Cytochrome P450 family 1 subfamily A member 1	130.59	0	0
	NM_000104	CYP1B1	Cytochrome P450 family 1 subfamily B member 1	22.18	0	0
	NM_001072	UGT1A6	UDP glucuronosyltransferase family 1 member A6	5.83	5.87×10^{-179}	2.36×10^{-175}
	NM_001242412	AHRR	Aryl-hydrocarbon receptor repressor	3.71	1.92×10^{-122}	5.15×10^{-119}
	NM_175617	MT1E	Metallothionein 1E	3.56	2.70×10^{-102}	4.81×10^{-99}
	NM_001270364	ALDH1L1	Aldehyde dehydrogenase 1 family member L1	2.98	3.89×10^{-37}	5.79×10^{-35}
	NM_000691	ALDH3A1	Aldehyde dehydrogenase 3 family member A1	2.91	3.77×10^{-20}	1.68×10^{-18}
	NM_005953	MT2A	Metallothionein 2A	2.81	3.10×10^{-72}	1.84×10^{-69}
	NM_015567	SLITRK5	SLIT and NTRK like family member 5	2.78	4.13×10^{-12}	6.85×10^{-11}
	NM_019093	UGT1A3	UDP glucuronosyltransferase family 1 member A3	2.48	2.99×10^{-8}	2.69×10^{-7}
	NR_027252	CYP1B1-AS1	CYP1B1 antisense RNA 1	2.47	1.87×10^{-7}	1.47×10^{-6}
	NM_176870	MT1M	Metallothionein 1M	2.12	1.73×10^{-5}	9.26×10^{-5}
	NM_005952	MT1X	Metallothionein 1X	2.11	9.78×10^{-22}	4.93×10^{-20}
	NM_000848	GSTM2	Glutathione S-transferase mu 2	2.05	1.00×10^{-11}	1.58×10^{-10}
	NM_001054	SULT1A2	Sulfotransferase family 1A member 2	2.02	2.33×10^{-13}	4.58×10^{-12}
	NM_000146	FTL	Ferritin light chain	2.00	3.49×10^{-45}	6.91×10^{-43}
	NM_000782	CYP24A1	Cytochrome P450 family 24 subfamily A member 1	−3.47	8.58×10^{-57}	2.65×10^{-54}
	NM_000779	CYP4B1	Cytochrome P450 family 4 subfamily B member 1	−3.09	1.89×10^{-91}	2.53×10^{-88}
	NM_001033044	GLUL	Glutamate-ammonia ligase (glutamine synthetase)	−2.85	2.60×10^{-11}	3.79×10^{-10}
	NM_018475	TMEM165	Transmembrane protein 165	−2.29	1.68×10^{-68}	8.17×10^{-66}
	NM_000765	CYP3A7	Cytochrome P450 family 3 subfamily A member 7	−2.27	1.97×10^{-7}	1.54×10^{-6}
	NM_005420	SULT1E1	Sulfotransferase family 1E member 1	−2.26	4.55×10^{-10}	5.48×10^{-9}

	NM_000689	ALDH1A1	Aldehyde dehydrogenase 1 family member A1	−2.21	9.16×10^{-12}	1.45×10^{-10}
	NR_144551	CYP2T1P	Cytochrome P450 family 2 subfamily T member 1, pseudogene	−2.04	2.74×10^{-12}	4.64×10^{-11}
	NM_001142776	CHAC1	ChaC glutathione specific γ-glutamylcyclotransferase 1	−2.04	2.14×10^{-12}	3.68×10^{-11}
Inflammatory response	NM_001302123	CXCL11	C-X-C motif chemokine ligand 11	2.56	3.76×10^{-12}	6.27×10^{-11}
	NM_000576	IL1B	Interleukin 1 β	2.48	3.20×10^{-12}	5.37×10^{-11}
	NM_001350888	AK7	Adenylate kinase 7	2.32	6.48×10^{-20}	2.82×10^{-18}
	NM_001066	TNFRSF1B	TNF receptor superfamily member 1B	2.17	2.24×10^{-12}	3.83×10^{-11}
	NM_000600	IL6	Interleukin 6	2.03	9.47×10^{-9}	9.34×10^{-8}
	NM_001190942	TNFSF10	TNF superfamily member 10	−3.95	8.12×10^{-136}	2.60×10^{-132}
	NM_003357	SCGB1A1	Secretoglobin family 1A member 1	−3.18	6.64×10^{-21}1	3.12×10^{-19}
	NM_004795	KL	Klotho	−3.10	8.08×10^{-64}	3.24×10^{-61}
	NM_001511	CXCL1	C-X-C motif chemokine ligand 1	−2.89	1.94×10^{-14}	4.38×10^{-13}
	NM_002546	TNFRSF11B	TNF receptor superfamily member 11b	−2.87	9.74×10^{-14}	2.01×10^{-12}E
	NM_002993	CXCL6	C-X-C motif chemokine ligand 6	−2.49	8.65×10^{-11}	1.17×10^{-9}
	NM_001291737	CD24	CD24 molecule	−2.38	1.33×10^{-118}	3.04×10^{-115}
	NM_001005474	NFKBIZ	NFκB inhibitor zeta	−2.23	2.18×10^{-49}	5.07×10^{-47}
	NM_144717	IL20RB	interleukin 20 receptor subunit beta	−2.20	1.16×10^{-24}	7.51×10^{-23}
	NM_003246	THBS1	Thrombospondin 1	−2.18	1.49×10^{-10}	1.93×10^{-9}
	NM_001319196	S100A8	S100 calcium binding protein A8	−2.03	1.57×10^{-6}	1.04×10^{-5}
Oxidative stress	NM_000857	GUCY1B3	Guanylate cyclase 1 soluble subunit beta	3.64	2.17×10^{-25}	1.51×10^{-23}
	NM_001123066	MAPT	Microtubule associated protein tau	2.38	1.43×10^{-10}	1.87×10^{-9}
	NM_001135241	AKR1C2	Aldo-keto reductase family 1 member C2	2.19	8.52×10^{-6}	4.87×10^{-5}
	NM_000962	PTGS1	Prostaglandin-endoperoxide synthase 1	2.16	1.25×10^{-28}	1.09×10^{-26}
	NM_001321015	GATM	Glycine amidinotransferase	2.11	2.36×10^{-23}	1.40×10^{-21}
	NM_000345	SNCA	Synuclein alpha	2.08	7.58×10^{-11}	1.03×10^{-9}
	NM_000878	IL2RB	Interleukin 2 receptor subunit beta	2.00	3.39×10^{-8}	3.02×10^{-7}
	NM_000930	plat	Plasminogen activator, tissue type (tPA)	−4.59	3.78×10^{-86}	3.57×10^{-83}
	NM_005066	SFPQ	Splicing factor proline and glutamine rich	−2.54	1.56×10^{-59}	5.33×10^{-57}
	NM_005252	FOS	Fos proto-oncogene, AP-1 transcription factor subunit	−2.36	3.31×10^{-19}	1.31×10^{-17}
	NM_001143818	SERPINB2	Serpin family B member	−2.07	5.01 ×	1.68 ×

			2		10^{-59}	10^{-56}
	NM_003356	UCP3	Uncoupling protein 3	−2.02	3.11×10^{-7}	2.33×10^{-6}
Lipid metabolism	NM_001142807	ACOXL	Acyl-CoA oxidase like	3.88	3.52×10^{-70}E-70	1.88×10^{-67}E-67
	NM_000527	LDLR	Low density lipoprotein receptor	3.18	3.98×10^{-78}	2.78×10^{-75}
	NM_174936	PCSK9	Proprotein convertase subtilisin/kexin type 9	2.93	3.21×10^{-60}	1.17×10^{-5}
	NM_001017369	MSMO1	Methylsterol monooxygenase 1	2.64	6.14×10^{-50}	1.49×10^{-47}
	NM_005063	SCD	Stearoyl-CoA desaturase	2.42	6.36×10^{-62}	2.37×10^{-59}
	NM_001076552	ACSS2	Acyl-CoA synthetase short-chain family member 2	2.36	5.06×10^{-87}	5.08×10^{-84}
	NM_001346590	INSIG1	Insulin induced gene 1	2.25	8.28×10^{-50}	1.96×10^{-47}
	NM_001163817	DHCR7	7-dehydrocholesterol reductase	2.16	3.18×10^{-76}	2.04×10^{-73}
	NM_001098272	HMGCS1	3-hydroxy-3-methylglutaryl-CoA synthase 1	2.01	1.09×10^{-8}	1.06×10^{-7}
	NM_000253	MTTP	Microsomal triglyceride transfer protein	2.01	8.67×10^{-5}	3.91×10^{-4}
	NM_005544	IRS1	Insulin receptor substrate 1	−3.20	3.85×10^{-39}	6.11×10^{-37}
	NM_000599	IGFBP5	Insulin like growth factor binding protein 5	−2.44	5.72×10^{-7}	4.10×10^{-6}
	NM_003561	PLA2G10	Phospholipase A2 group X	−2.29	2.37×10^{-12}	4.05×10^{-11}
	NM_001172895	CAV1	Caveolin 1	−2.13	4.93×10^{-44}	9.32×10^{-42}
	NM_001285829	CEBPA	CCAAT/enhancer binding protein alpha	−2.01	1.33×10^{-19}	5.53×10^{-1}
Cell adhesion and cell barrier	NM_001254750	CD6	CD6 molecule	2.75	7.43×10^{-11}	1.01×10^{-9}
	NM_001252335	CGNL1	Cingulin like 1	2.25	7.36×10^{-24}	4.58×10^{-22}
	NM_020884	MYH7B	Myosin heavy chain 7B	2.00	4.81×10^{-9}	4.97×10^{-8}
	NM_001185056	CLDN11	Claudin 11	−3.48	2.19×10^{-36}	3.06×10^{-34}
	NM_001001890	RUNX1	Runt related transcription factor 1	−2.38	1.97×10^{-72}	1.22×10^{-69}
	NM_000492	CFTR	Cystic fibrosis transmembrane conductance regulator	−2.31	8.48×10^{-20}	3.64×10^{-18}
	NM_001795	CDH5	Cadherin 5	−2.23	4.31×10^{-57}	1.36×10^{-54}
	NM_001308176	CDH2	Cadherin 2	−2.15	1.66×10^{-12}	2.90×10^{-11}
	NM_001271899	PPP2R2B	Protein phosphatase 2 regulatory subunit Bbeta	−2.14	9.85×10^{-16}	2.67×10^{-14}
	NM_198722	AMIGO3	Adhesion molecule with Ig like domain 3	−2.14	5.85×10^{-16}	1.61×10^{-14}
	NM_001253849	VTCN1	V-set domain containing T-cell activation inhibitor 1	−2.14	3.19×10^{-82}	2.56×10^{-79}
	NM_014141	CNTNAP2	Contactin associated protein-like 2	−2.09	1.69×10^{-23}	1.01×10^{-21}
	NM_021101	CLDN1	Claudin 1	−2.08	2.02×10^{-67}	9.25×10^{-65}
